# Single and Double Strand Sperm DNA Damage: Different Reproductive Effects on Male Fertility

**DOI:** 10.3390/genes10020105

**Published:** 2019-01-31

**Authors:** Jordi Ribas-Maynou, Jordi Benet

**Affiliations:** Unitat de Biologia Cel·lular i Genètica Mèdica, Departament de Biologia Cel·lular, Fisiologia i Immunologia, Facultat de Medicina, Universitat Autònoma de Barcelona, 08193 Bellaterra, Spain

**Keywords:** sperm DNA damage, DNA fragmentation, infertility, assisted reproduction, miscarriage, implantation

## Abstract

Reproductive diseases have become a growing worldwide problem and male factor plays an important role in the reproductive diagnosis, prognosis and design of assisted reproductive treatments. Sperm cell holds the mission of carrying the paternal genetic complement to the oocyte in order to contribute to an euploid zygote with proper DNA integrity. Sperm DNA fragmentation had been used for decades as a male fertility test, however, its usefulness have arisen multiple debates, especially around Intracytoplasmic Sperm Injection (ICSI) treatments. In the recent years, it has been described that different types of sperm DNA breaks (single and double strand DNA breaks) cause different clinical reproductive effects. On one hand, single-strand DNA breaks are present extensively as a multiple break points in all regions of the genome, are related to oxidative stress and cause a lack of clinical pregnancy or an increase of the conception time. On the other hand, double-strand DNA breaks are mainly localized and attached to the sperm nuclear matrix as a very few break points, are possibly related to a lack of DNA repair in meiosis and cause a higher risk of miscarriage, low embryo quality and higher risk of implantation failure in ICSI cycles. The present work also reviews different studies that may contribute in the understanding of sperm chromatin as well as treatments to prevent sperm DNA damage.

## 1. Introduction

Different fertility societies around the globe and the World Health Organization estimate that infertility is present in between 7% and 15% of couples in reproductive age [1,2]. In a high number of cases female factors and especially female age [3], are the most important causes of infertility, however, different male factors are present in at least 50% of the couples presenting this disorder [4]. Due to the high percentage of incidence in the pathology, recent research suggests that sperm cell and sperm DNA may have a major influence not only in natural conception but also in fertility treatments [5,6]. 

In front of a fertility disorder or a fertility treatment, microscopic semen analysis measuring sperm concentration, motility and morphology has been the traditional and important first approach to male infertility and, although a high decrease of these parameters had been associated to a lack of achievement of natural pregnancy [7] and nowadays home-based technologies in order to advance the first diagnosis are emerging [8]. However, in most cases these parameters are not indicative of the positive performance of assisted reproduction techniques (ART) [5,9]. In fact, although they are improving, ICSI treatments reached limited implantation rates [10]. Because of that, a deeper study is necessary in most cases to elucidate the alteration in order to design the best treatment in each case. 

## 2. Sperm DNA and Sperm DNA Damage

**Figure 1:**
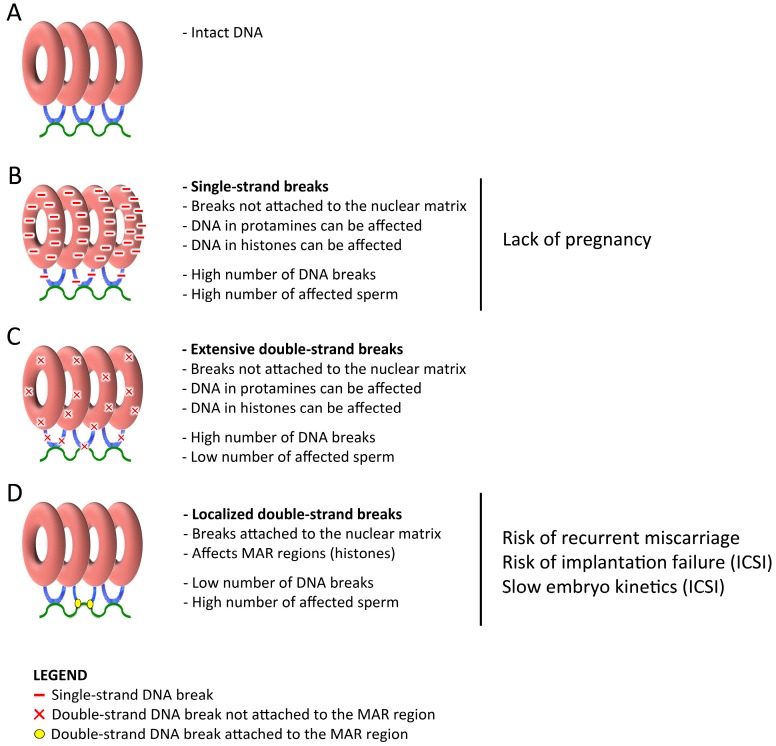
Schematic structure of the sperm DNA compacted in protamines that form toroid structures (red) linked by MAR regions (matrix attachment regions) compacted in histones (blue) and attached to the nuclear matrix (green). (**A**) represents an intact chromatin. (**B**) represents chromatin with single-strand breaks (red lines). (**C**) represents chromatin with extensive double-strand breaks (red cross). (**D**) represents chromatin with localized double-strand breaks attached to the nuclear matrix (yellow circle).

Spermatogenesis is a very complex cellular process that implies both meiosis and cell differentiation. The main stage of meiosis is in prophase I where, spermatocytes deliberately produce double-strand DNA breaks (DSB) through Spo11 protein [11,12]. These DSB are necessary for homologous chromosomes to allow DNA recombination. Then, after strand invasion, DSB activate the DNA repair machinery through the protein kinase ataxia-telangiectasia mutated (ATM) in order to repair the free ends and therefore generate the chiasma by homologous recombination and ATM is also responsible of inhibiting the formation of new DSB by Spo11 [12,13]. After meiosis, haploid round spermatids suffer a cell differentiation, loosing most part of their cytoplasm and acquiring midpiece and flagellum in order to possess motility after ejaculation [14]. However, in terms of chromatin, the most important change happening in spermatids is the exchange of histones by protamines, which extraordinarily compact about 85% of the human sperm DNA in toroidal structures tied between them and bond to the nuclear matrix by the matrix attachment regions (MAR regions) (Figure 1). These MAR regions remain compacted by histones and represent a very small part of the genome estimated to be around 15% of the human sperm chromatin [15,16]. This high-grade of DNA compaction with protamines, coupled to a motile architecture of the cell, give the sperm the perfect features to carry male genetic material to oocyte to form the zygote. It is obvious that if this male genetic material contains alterations, these may affect the zygote somehow [17]. In fact, it is undeniable that DNA breaks induce a cellular response in somatic cells leading to an activation of DNA repair machinery, apoptosis or cell transformation, being the basis of cancer and other diseases [18,19]. Different works in embryos analysing the effect of induced DNA breaks in animal sperm cells through radiation observed multiple chromosomal alterations such as chromosome breaks, translocations, fusions and acentric fragments in the zygote [17,20]. 

In the last decade, the previous evidences suggested the incorporation of the sperm DNA fragmentation tests as a promising analysis in male reproduction and multiple studies were performed in the field since then [21]. Regarding natural conception, multiple works show a relation of sperm DNA fragmentation (SDF) to a lack of clinical pregnancy and an increase of time of conception [22,23,24]. However, after ICSI procedures, opposite results were found by different research groups regarding embryo quality, implantation and pregnancy outcomes, being some studies that show a positive relation of SDF [25,26,27,28] and others that show a negative relation of SDF to clinical outcomes [29,30,31,32,33]. This controversy, coupled that only a few studies were conducted in a prospective and double blind manner, led the American Society for Reproductive Medicine to refuse its routine use in 2013 [34]. However, some promising results arisen in the last years might be the explanation why the traditionally measured sperm DNA damage present a lack of predictive power in ICSI.

The debate in sperm DNA fragmentation started regarding which of all DNA analysis techniques, that rely on different mechanisms for DNA breaks detection, was the best for the male infertility diagnosis. Understanding the basis of each technique and the correlations between them is critical to understand their implications in the male fertility diagnosis and to compare between them. Techniques are explained in the following part of the review and are summarized in Table 1.

On one hand, the most used techniques for the analysis of sperm DNA fragmentation have traditionally been the Terminal deoxynucleotidyl transferase dUTP nick end labelling (TUNEL), Sperm Chromatin Structure Assay (SCSA) and Sperm Chromatin Dispersion (SCD) test. These techniques offer a unique value of sperm with DNA fragmentation, independently of the type (single and double-strand DNA breaks) and the region (toroids compacted in protamines or MAR regions compacted in histones).

TUNEL assay [35] relies on a terminal TdT transferase for the labelling of 3′ free ends of DNA, resulting in a higher labelling on fragmented sperm cells. Different modifications have been introduced in the protocol in order to increase its sensitivity in sperm cells, such as the use of a previous DNA decompaction using dithiothreitol (DTT) or the use of flow cytometer [36,37,38].

SCSA is based on an acid denaturation of the chromatin and staining with acridine orange. When DNA breaks are present, chromatin is more susceptible to denaturation and acridine orange accumulates in the DNA emitting in red fluorescence. When DNA breaks are not present, acridine orange intercalates in the double helix and emits in green fluorescence. Fluorescence is captured using a cytometer in order to determine DNA fragmentation [39].

SCD test uses a sperm lysis solution based on DTT, sodium dodecyl sulphate (SDS) and NaCl to remove the sperm membrane and protamines, that causes the formation of DNA haloes, which allow the differentiation of fragmented and non-fragmented sperm cells [40]. 

**Figure 2:**
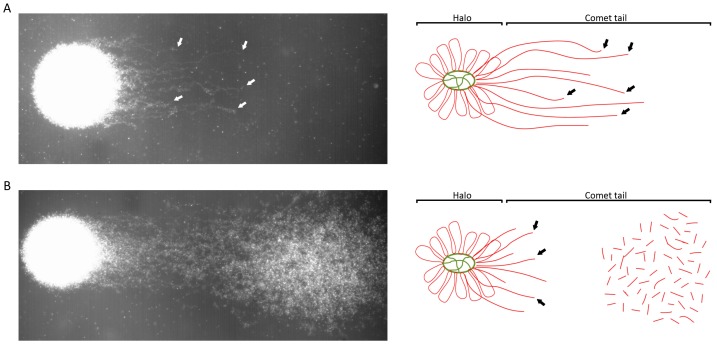
(**A**) Picture and scheme of neutral Comet with localized DSB (double-strand DNA breaks) attached to the nuclear matrix (green). Comet halo consists in non-fragmented chromatin and comet tail is formed by chromatin fibres attached to the nuclear matrix with low number of DNA breaks at the end (arrows). (**B**) Picture and scheme of neutral Comet with extensive DSB. Comet tail is formed by DNA fragments that are not attached to the nuclear matrix. This comet also shows part of localized DNA breaks attached to the MAR region (arrow).

On the other hand, Comet assay [41] relies on a DNA decompaction and protein depletion coupled to a single-cell electrophoresis in an agarose micro gel. DNA molecules that contain breaks move towards the cathode and the length of the “comet tail” can be measured to determine the grade of DNA fragmentation at a single cell level. This technique has been applied in multiple different protocols, which usually vary in agarose concentrations and in electrophoresis times [42,43]. As the Comet assay can be performed in alkaline or neutral pH, different types of DNA breaks can be detected (Table 1) (Figure 1): (i) alkaline Comet assay performed in a small electrophoresis time (about four minutes) detect mostly single-strand DNA breaks affecting both toroidal regions and MAR regions in a high number of break points [44,45] and (ii) neutral Comet assay can detect two types of double-strand DNA breaks (Figure 2): (a) extensive DSB, which represent a very small part of total DSB and can be observed as very long comet tails separated from the sperm core; and (b) localized DSB localized and attached to the MAR region, as demonstrated in pulsed-field gel electrophoresis [43,44,45,46], being the most common DSB. Although extensive DSB result in longer Comet tails, they cannot be distinguished from localized DSB in a single Comet. However, when a semen sample present high number of sperm cells with extensive DSB (long tails), single-strand DNA damage is also present in a high amount (Ribas-Maynou personal observation). Previous studies had shown that localized DSB represent very few break points in the genome, as long chromatin fibres with a break point in the end can be seen in a detailed neutral Comet image (Figure 2A), which is supported by Kaneko et al., using pulsed field gel electrophoresis [47]. We demonstrated that localized DSB remain attached to the sperm nuclear matrix [45], maybe through a TOP2B or similar protein [45,46], a very important feature taking into account that the nuclear matrix is inherited to the male pronucleus in the zygote [46,48,49,50], giving a chance to the embryo to repair the DSB.

Studies using all the techniques showed that oxidative damage detected by alkaline Comet assay presented a good correlation to TUNEL, SCSA and SCD techniques [23,51,52]. Although these techniques may potentially detect double-strand breaks, a study conducted by our group analysing the same semen samples with five methodologies showed that no correlation was present with the neutral Comet assay [23]. Then, the latter would be the only technique that is able to differentially detect MAR-region double-strand breaks [23,44], whereas TUNEL, SCSA and SCD may detect extensive DSB. A Comet assay variant (two-tailed Comet assay) applying both alkaline and neutral Comet assay in the same slide by turning it 90º between electrophoresis allows to distinguish single and double-strand DNA breaks on the same sperm cell [53]. However, no studies have been performed comparing these techniques and alkaline or neutral Comet assay separately in order to elucidate if double-strand breaks detected in two-tailed Comet assay correspond to MAR region localized DSB.

## 3. Oxidative DNA Damage, Alkaline Comet Assay and Pregnancy Achievement

Using alkaline Comet assay in different cohorts, an study published in 2012 [43] showed that the extensive single-strand DNA breaks were reversely associated to the achievement of natural pregnancy independently of the neutral Comet results (Figure 1 and Table 1). This was confirmed and compared with TUNEL, SCSA and SCD tests in 2013, demonstrating also that alkaline Comet is the most sensitive technique for the prediction of natural pregnancy achievement [23,43]. Which is also in accordance to the numerous studies from other research groups that find similar association in natural pregnancy using TUNEL, SCSA, SCD and Comet assay tests [5,51,54,55,56,57,58].

Single-strand breaks are produced mainly due to reactive oxygen species (ROS) [42,53,59], which may come from exogenous sources such as environmental toxicants, smoking, alcohol, diet, radiation and so forth or from endogenous sources such as an increase of leukocytes, presence of varicocele or even the ROS generated by mitochondria for the movement of sperm cell [60,61,62]. Free radicals may cause lipid peroxidation, mitochondrial and nuclear DNA base modifications such as 8-OH-guanine and 8-OH-2′-deoxyguanosine (8-OHdG), an oxidized base adduct that destabilize DNA structure and cause a DNA break [63,64,65]. This affectation does not find a restriction by DNA condensation and therefore may affect both toroids compacted in protamines and MAR regions compacted in histones [44]. Then, if such an extensive damage happens to the sperm DNA due to oxidative stress, the sperm membranes would also be affected and usually sperm motility is lost. Because of that, a strong negative relation between progressive motility and oxidative damage (single-strand DNA damage) analyzed using TUNEL, SCSA, SCD and alkaline Comet [55,61,66].

As mentioned before in this review, controversial results are found in different studies regarding ICSI outcomes: some of them which found predictive value of oxidative damage [25,26,27,28] and other with opposite results [29,30,31,32,33]. If single-strand DNA breaks present a correlation to progressive motility and sperm morphology and ICSI procedures use the most motile sperm cells with better morphology, paternal genome should be free of oxidative damage. In this regard, a work by Gosalvez et al. [67] demonstrated that motile sperm organelle morphology examination (MSOME) selected sperm cells were free of DNA damage analysed by SCD test. Moreover, a work using Comet assay suggested that grade I and II sperm cells present lower incidence of oxidative DNA damage than grade III and IV [68]. These results need to be further confirmed in conventional ICSI sperm selection. However, our data suggest that no relation is present between alkaline Comet and embryo quality, embryo kinetics or implantation [69].

## 4. Double-Strand DNA Damage, Recurrent Miscarriage and Preimplantation Failure in ICSI Cycles

Analysing the data of the patients and donors with high DSB, a specific profile was observed with low oxidative damage and high neutral comet values in patients with first trimester recurrent miscarriage where all related female factors were discarded and in one subgroup of fertile donors [44]. In a recent study, our group has found that patients with this profile who undergo ICSI treatments produce embryos with a delayed embryo development to blastocyst, which also cause lower implantation rates [69]. Other works also show that double-strand breaks may contribute to a higher implantation failure risk [6,25]. Since implantation failures in ICSI cycles and miscarriages present similar profiles with high DSB, one may think that they might have similar origin. In fact, small number of DNA breaks localized in concrete regions of the genome might induce a cell failure where the affected regions are necessary for the development. In our last study, embryos that achieved implantation presented faster embryo kinetics than those that did not achieve implantation [69]. In fact, faster embryo kinetics had been associated to embryo euploidy [70,71,72].

DSB are the most lethal alteration that may happen in a zygote, since paternal and maternal pronucleus remain separated in early mammalian embryos and, therefore, no complementary chain would be available for DNA repair [73,74,75] and a few number of DSB are sufficient to delay cell cycle [76]. It is important to note that paternal double-strand breaks remain attached to the nuclear matrix and probably to other proteins such as TOP2B [20,46,77] and the nuclear matrix is inherited at male pronucleus until first mitotic division [49,78]. This may be crucial at the zygote, because it may give a chance to correctly repair both free ends of the double-strand break. There is a consensus point that oocyte quality may play a role in this DNA repair, since different studies proved that early embryos are able to repair DNA damage [79,80,81,82,83,84]. In this sense, in patients with DSB, the most significant delay observed in the embryo kinetics was just after fertilization, indicating that DNA repair machinery may be active in this stage [69]. Recent studies in sperm cells demonstrated that MAR regions are required as a scaffold for DNA replication after fertilization [48] and, in somatic cells, nuclear matrix also is involved in transcription, cell regulation and replication [85,86]. In mammals, inducing DSB in sperm cells and used these sperm cells to fertilize eggs observed chromosomal alterations in paternal genome of the embryo and showing also a delay in the first embryo cleavage [17,20,87]. Moreover, studies inducing double-strand DNA breaks in mice sperm through radiation observed a p53 and p21 related response and less number of foetuses [88,89] or less survival of offspring in a dose dependent manner [90].

## 5. Prevention of DNA Damage

The data presented in the studies referenced before supports that oxidative damage may affect the pregnancy achievement capacity due to misbalanced levels of oxidants/antioxidants [61,91]. 

The use of antioxidants has been widely applied in subfertile males [92]. Several works demonstrated that they are a positive contribution on sperm count, motility, morphology and also proved that they help reducing oxidative DNA fragmentation [93,94,95,96]. Although there are very few studies with randomized and placebo controls, Cochrane review suggests that the use of antioxidants causes from 1.8 to 4.6 fold increase in the chances of achieving a natural pregnancy. However, up to a 6.5 fold increase in miscarriages might be observed [97]. In ICSI treatments, it is still not clear if antioxidants could help on improving pregnancy and birth rates [98,99,100]. High quality studies including different groups of patients are necessary in order to elucidate the need of antioxidants in ICSI procedures. 

Treatments for the reduction of double-strand sperm DNA damage should also reduce the miscarriage risk and the implantation failure risk in ICSI cycles, showing also less delay on embryo kinetics. Until our knowledge, no validated treatment reduce the incidence of MAR-region localized DSB. However, a study conducted in humans in 2006 by Schmid and colleagues demonstrated that men with daily caffeine consumption presented increased values of DSB measured with neutral Comet independently of male age in healthy non-smokers [101]. Caffeine is a known inhibitor of DNA repair, as it has been described that inhibits ATM kinase [102,103] and DNA resection in homologous recombination through Rad51 [104,105]. Also, it has been reported to affect cell cycle at both G1/S and G2/M checkpoints and inducing programmed cell death through p53-dependent pathway [106]. Studies in animals reported that caffeine administration to rats caused an impairment of pregnancy [107]. Other studies inducing DNA strand breaks in sperm cells through radiation and cultivating the oocytes and the produced embryos in caffeine demonstrated that chromosome and chromatid aberrations persist in the zygote, indicating oocyte DNA repair is inhibited by caffeine [17]. Since spermatocytes must produce double-strand breaks through Spo11 in prophase I in order to perform DNA recombination and later, they need to repair these DSB. According to previous results, the consumption of caffeine would impair ATM kinase and/or resection of double-strand breaks [104,105] and may induce that a few double-strand breaks would not be repaired, causing that mature sperm cells present DSB [101]. Further basic studies are needed to explain how a spermatocyte with double-strand breaks can escape the pachytene checkpoint [108,109]. Reducing the incidence of DSB in sperm cell would improve clinical outcomes in terms of miscarriage and implantation in ICSI cycles.

## Figures and Tables

**Table 1:** Techniques for the detection of different types of DNA damage.

Technique	Basic Principle	Advantages	Disadvantages	Type of DNA Damage Detected	Clinical Effect
TUNEL	Labelling of 3′ free ends with a TdT transferase. Breaks are directly labelled.	· Highly standardized protocol.	· Need of flow cytometer for higher number of analysed cells. · Sensitivity for the detection of DNA breaks in sperm cells. · No detection of MAR-region attached DSB.	· Single-strand breaks. · Extensive DSB.	· Pregnancy achievement.
SCSA	Acid denaturation followed by staining with Acridine Orange. DNA with breaks is more susceptible to denaturating.	· Standardized and fast protocol. · Differentiation of immature sperm cells (HDS%).	· Need of flow cytometer. · No detection of MAR-region attached DSB.	· Single-strand breaks. · Extensive DSB.	· Pregnancy achievement.
SCD	Acid denaturation, lysis of sperm membranes and extraction of protamines using detergent and salt. Non-fragmented sperm cells form a halo and fragmented sperm cells do not form halo (form a huge halo that cannot be seen at the optic microscope)	· Highly standardized protocol.	· Non-standardized analysis. · Number of analysed sperm cells · No detection of MAR-region attached DSB.	· Single-strand breaks. · Extensive DSB.	· Pregnancy achievement.
Alkaline Comet	Lysis of sperm membranes and extraction of protamines, alkaline denaturation and electrophoresis at alkaline pH. DNA breaks migrate towards cathode forming a DNA tail.	· Differentiation of mostly single strand DNA breaks at 4 minutes of electrophoresis. · Modulation: longer electrophoresis time may allow elucidating total DNA damage. · Allow quantification of DNA breaks with specific software.	· Technique and analysis are not standardized between laboratories. · No detection of MAR-region DSB. · Studies comparing different electrophoresis times are needed.	· Mostly single-strand breaks (4 min. electrophoresis). · Probably extensive DSB.	· Pregnancy achievement (4 min. electrophoresis time). · Some studies related alkaline Comet to ICSI success using longer times of electrophoresis.
Neutral Comet	Lysis of sperm membranes and extraction of protamines and electrophoresis at neutral pH. DNA breaks migrate towards cathode forming a DNA tail.	· Differentiation of MAR-region specific DSB.	· Technique and analysis are not standardized between laboratories.	· MAR-region specific double strand breaks. · Extensvie DSB.	· First trimester miscarriage risk. · Risk of implantation failure in ICSI cycles. · May be associated to slower embryo kinetics.
Two-tailed Comet	Lysis of sperm membranes and extraction of protamines. First, neutral electrophoresis and, after alkaline denaturation and rotation of slide 90º, alkaline electrophoresis. Sperm present two DNA tails.	· Detection of single and double strand DNA breaks in the same sperm cell.	· Technique not standardized · Difficult interpretation. Requires experienced observer.	· Single-strand breaks. · Extensive DSB. · Not known if MAR-region specific double strand breaks (lack of studies comparing to neutral Comet alone).	· Pregnancy achievement. · Need of human clinical studies regarding ICSI.

HDS: High DNA Stainable sperm; TUNEL: Terminal deoxynucleotidyl transferase dUTP nick end labelling; SCSA: Sperm Chromatin Structure Assay; SCD: Sperm Chromatin Dispersion; ICSI: Intracytoplasmic sperm injection.
